# Diagnosis of soil-transmitted helminths using the Kato-Katz technique: What is the influence of stirring, storage time and storage temperature on stool sample egg counts?

**DOI:** 10.1371/journal.pntd.0009032

**Published:** 2021-01-22

**Authors:** Felix Bosch, Marta S. Palmeirim, Said M. Ali, Shaali M. Ame, Jan Hattendorf, Jennifer Keiser

**Affiliations:** 1 Swiss Tropical and Public Health Institute, Basel, Switzerland; 2 University of Basel, Basel, Switzerland; 3 Public Health Laboratory Ivo de Carneri, Chake Chake, United Republic of Tanzania; University of Cambridge, UNITED KINGDOM

## Abstract

**Background:**

Soil-transmitted helminths infect about one fifth of the world’s population and have a negative impact on health. The Kato-Katz technique is the recommended method to detect soil-transmitted helminth eggs in stool samples, particularly in programmatic settings. However, some questions in its procedure remain. Our study aimed to investigate the effect of storage time, storage temperature and stirring of stool samples on fecal egg counts (FECs).

**Methodology/Principal findings:**

In the framework of a clinical trial on Pemba Island, United Republic of Tanzania, 488 stool samples were collected from schoolchildren. These samples were evaluated in three experiments. In the first experiment (n = 92), two Kato-Katz slides were prepared from the same stool sample, one was stored at room temperature, the other in a refrigerator for 50 hours, and each slide was analyzed at nine time points (20, 50, 80, 110, 140 minutes, 18, 26, 42 and 50 hours). In the second experiment (n = 340), whole stool samples were split into two, one part was stored at room temperature, and the other part was put in a refrigerator for 48 hours. From each part one Kato-Katz slide was prepared and analyzed at three time points over two days (0, 24 and 48 hours). In the third experiment (n = 56), whole stool samples where stirred for 15 seconds six times and at each time point a Kato-Katz slide was prepared and analyzed.

Mean hookworm FECs of Kato-Katz slides stored at room temperature steadily decreased following slide preparation. After two hours, mean hookworm FECs decreased from 22 to 16, whereas no reduction was observed if Kato-Katz slides were stored in the refrigerator (19 vs 21). The time x storage interaction effect was statistically significant (coefficient 0.26, 95% CI: 0.17 to 0.35, p < 0.0001). After 24 hours mean hookworm FECs dropped close to zero, irrespective of the storage condition. Whole stool samples stored at room temperature for one day resulted in a mean hookworm FEC decrease of 23% (p < 0.0001), compared to a 13% reduction (p < 0.0001) if samples were stored in the refrigerator. Fecal egg counts of *A*. *lumbricoid*e*s* and *T*. *trichiura* remained stable over time regardless of storage temperature of whole stool samples. Finally, we found a significant reduction of the variation of hookworm and *T*. *trichiura* eggs with increasing rounds of stirring the sample, but not for *A*. *lumbricoides*. For hookworm we observed a simultaneous decrease in mean FECs, making it difficult to draw recommendations on stirring samples.

**Conclusions/Significance:**

Our findings suggest that stool samples (i) should be analyzed on the day of collection and (ii) should be analyzed between 20–30 minutes after slide preparation; if that is not possible, Kato-Katz slides can be stored in a refrigerator for a maximum of 110 minutes.

## Author summary

Intestinal worms, such as hookworm, *Ascaris lumbricoides*, and *Trichuris trichiura*, infect one in five people worldwide. In children, they can cause cognitive and physical impairment. Accurate detection of these infections is important to treat patients and control the spread of intestinal worms. Currently, the Kato-Katz technique is the most commonly used diagnostic method, particularly, in programmatic settings. However, there are still several open questions to be resolved. In this study, we aimed at exploring the effect of storage time, storage temperature, and stirring of stool samples on the number of worm eggs detectable by the Kato-Katz technique. We included 488 stool samples collected from schoolchildren on Pemba Island, United Republic of Tanzania. We found that, at room temperature, hookworm eggs rapidly disappear from Kato-Katz slides, but storing them in a refrigerator preserves eggs for up to 110 minutes. For *A*. *lumbricoides* and *T*. *trichiura* no relevant trend in fecal egg counts in Kato-Katz slides over time and or influence of storing temperature was detected. However, storing whole stool samples in a refrigerator overnight did not prevent hookworm eggs from disappearing, thus, we recommend analyzing all samples on the day of collection. For *A*. *lumbricoid*e*s* and *T*. *trichiura* fecal egg counts remained stable in whole stool samples over time, regardless of storage temperature. Finally, we found that stirring samples reduced the variation of fecal egg counts for hookworm and *T*. *trichiura*, but not for *A*. *lumbricoides*. Hookworm fecal egg counts decreased with increasing rounds of sample stirring.

## Introduction

Globally, soil-transmitted helminths (STH) infect about 1.5 billion people and are widespread in tropical and sub-tropical regions [1]. Humid soil, inadequate sanitation, and personal hygiene, limited access to clean water, crowded living conditions, no shoe-wearing, lack of access to health care and health education are factors that favor the transmission of these parasites [1–4]. The most prevalent STHs are *Ascaris lumbricoides*, *Trichuris trichiura*, and hookworm (*Necator americanus*, *Ancylostoma duodenale* and *Ancylostoma ceylanicum*). Worldwide, an estimated 819 million people are infected with *A*. *lumbricoides*, 465 million with *T*. *trichiura* and 489 million with hookworm [5]. Soil-transmitted helminthiasis causes considerable morbidity with an estimated 1.9 million disability-adjusted life-years in 2017 [6]. Consequences of these infections include blood loss, iron deficiency anemia, and impairment of nutrient intake, digestion, and absorption. This can lead to cognitive and physical growth impairment in children [1,4,7].

Proper diagnosis of these infections is key to combat them. The Kato-Katz technique is the most common diagnostic approach for STH infections [8–11]. This method is recommended by the World Health Organization (WHO) due to its simplicity and relatively low cost, since most of the equipment is reusable. The Kato-Katz method shows good performance, particularly at detecting helminth infections of moderate and heavy intensities, and it seems to be equally sensitive as new diagnostic microscope-based methods such as McMaster, Mini-FLOTAC and FECPAK^G2^ or molecular methods as qPCR [2,9,12–17]. However, there is one major downside to this technique: its sensitivity to light infections is quite low [9,10,13,14,18–21]. The detection of eggs can be particularly difficult in the case of hookworm for several reasons. The thin-shelled ova of hookworm hatch in the soil soon after excretion and are less resistant than those of *A*. *lumbricoides* and *T*. *trichiura*, which only hatch after passage through the stomach [22]. Several studies have reported that the time between the stool sample collection and the laboratory processing significantly decreases hookworm fecal egg counts (FECs) [18,20,22,23]. When a large number of samples is collected and brought into the laboratory these might be stored overnight and analyzed the next day, due to time restrictions.

A second factor that might influence the sensitivity of Kato-Katz is related to inter-specimen and intra-specimen variation of helminth egg output, *i*.*e*. clustering of eggs within different parts of the stool sample [10,20,23–29]. To overcome intra-specimen and day-to-day variation in egg excretion, analyzing multiple Kato-Katz thick smears from a single and from multiple stool specimens collected over consecutive days is suggested [10,11,18,20,30]. Furthermore, it is recommended to homogenize the whole stool sample by stirring it well prior to preparing the Kato-Katz thick smear to control intra-specimen variation [18,22,31], since STH eggs might be patchily distributed [20,23,24,26,27,29]. Moreover, only a very small amount of stool (41.7 mg) is used for each Kato-Katz thick smear, making it even more likely to miss light intensity STH infections if the eggs are not evenly distributed in the sample [18,21]. However, studies have found no decrease in hookworm, *A*. *lumbricoides* and *T*. *trichiura* intra-sample variance of FECs after stirring the stool sample [23,24]. It is important to note that all these studies had small sample sizes ranging from two to nine individual stool samples.

A third reason why the sensitivity of Kato-Katz for hookworm is lower than for other parasites could be the time span between the preparation and reading of slides. Several studies have found [28] or hypothesized [2,9,26] that a delay significantly lowers the sensitivity and FECs for hookworm. The glycerol used in the Kato-Katz method destroys hookworm eggs over time, leading to a rapid clearing of eggs and making them invisible to the slide reader [21]. Therefore, the WHO recommends to microscopically analyze Kato-Katz thick smears within 30 to 60 minutes after slide preparation [17]. In contrast to hookworm eggs, *T*. *trichiura* and *A*. *lumbricoides* eggs are visible for several months [17]. However, it is not clear exactly how much time hookworm eggs remain visible in the Kato-Katz slide and whether different storing conditions could influence this. To our knowledge, no studies measuring the effect of different storing conditions overnight on STH FECs have been conducted. Furthermore, there are still no recommendations on how long one should stir a stool sample to achieve good homogenization of STH eggs.

It is, therefore, fundamental to further investigate how to optimize and increase the sensitivity of the Kato-Katz method, since otherwise the prevalence of infections can be systematically underestimated and, in the context of a clinical trial, cure rates, egg reduction rates and drug efficacy can be overestimated. Our study had the following two main objectives: (i) to quantify the impact of storage time and storage temperature on FECs of single Kato-Katz slides and whole stool samples, and (ii) to quantify the impact of homogenizing stool samples on the variation of FECs.

## Methods

### Ethics statement

This study was embedded in a school-based, randomized, open-label clinical trial conducted from July to September 2019 in four schools on Pemba Island, United Republic of Tanzania. The primary objective of the clinical trial was to evaluate the safety and efficacy of a new chewable formulation of mebendazole *versus* the standard swallowable tablet of mebendazole in children aged 3 to 12 years. The methodology and results of this clinical trial (registered at ClinicalTrials.gov number NCT03995680) are reported elsewhere [32].

### Study area and field procedures

For this study, a subset of the stool samples collected for the clinical trial were used. Participants received an empty stool container at school that they were asked to return the following morning with a stool sample. Samples were collected in schools, stored in boxes cooled with ice packs and transported by car to the central laboratory in Chake Chake, Pemba (Public Health Laboratory Ivo de Carneri).

### Laboratory procedures

To assess if time and storage temperature affected parasite FECs we performed two experiments: the first using the Kato-Katz slides and, the second using the whole stool samples prior to slide preparation. The third experiment aimed at assessing the influence of stirring stool samples on FECs. For all experiments, we selected samples from children we already knew were infected with at least one STH (hookworm, *A*. *lumbricoides*, and *T*. *trichiura*).

#### Kato-Katz storing experiment

**Fig 1:**
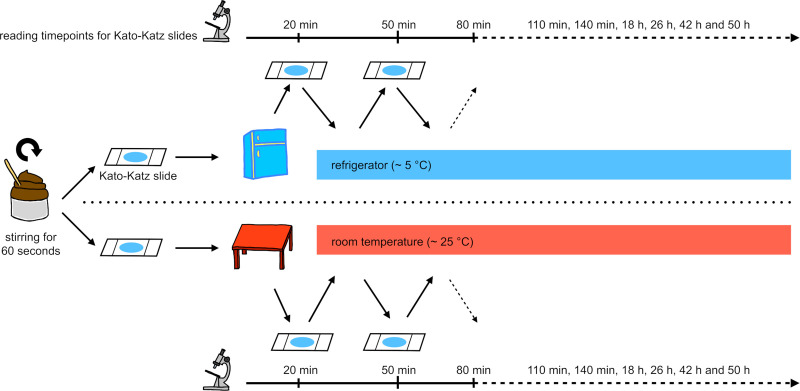
Procedure of the Kato-Katz storing experiment.

Stool samples selected for this experiment were stirred with a wooden spatula for one minute for homogenization. From each sample, two Kato-Katz thick smears were prepared using standard 41.7 mg templates. One of the slides was immediately stored in the refrigerator (~ 5°C) and the other one was stored at room temperature (~ 25°C). Twenty minutes later, both Kato-Katz slides were microscopically analyzed for the first time. Experienced laboratory technicians counted and recorded hookworm, *A*. *lumbricoides* and *T*. *trichiura* eggs. These same slides were then returned to their original storing conditions: room temperature or refrigerator. Slides were re-read at 50, 80, 110, and 140 minutes, as well as in the morning and evening of the two following days at 18, 26, 42, 50 hours. These slides were always kept either at room temperature or in the refrigerator between each reading time point (Fig 1).

#### Whole sample storing experiment

**Fig 2:**
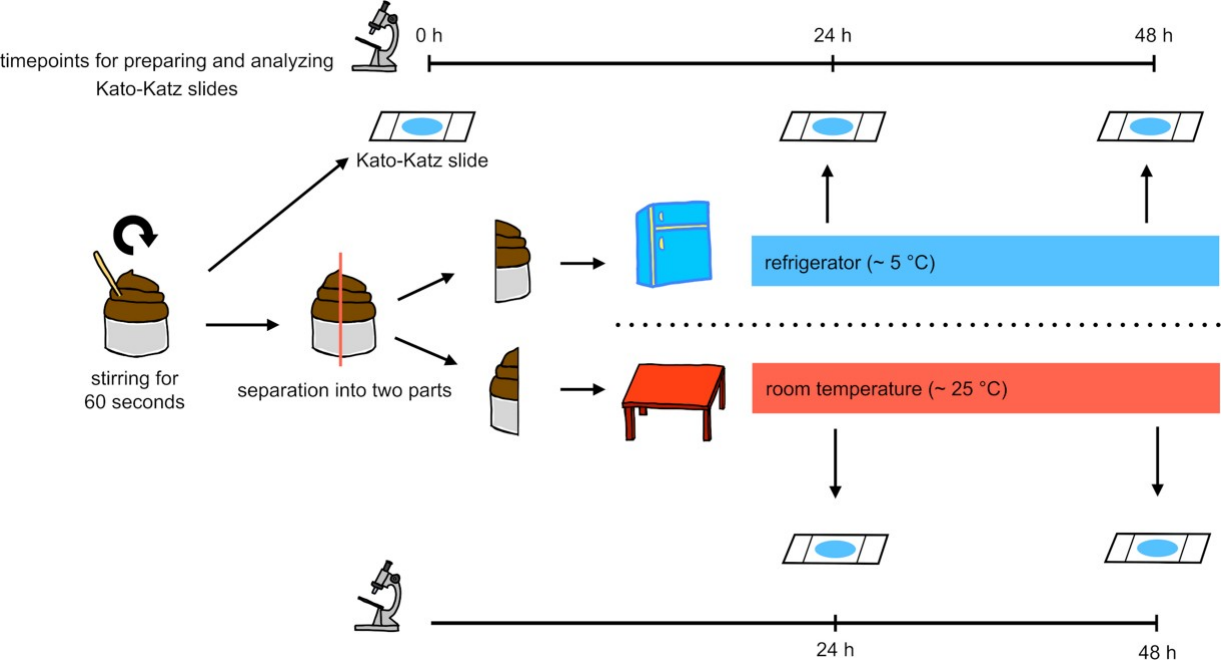
Procedure of the whole sample storing experiment.

Stool samples selected for this experiment were homogenized for one minute. A Kato-Katz slide was prepared from each of these samples and analyzed under a light microscope for hookworm, *A*. *lumbricoides*, and *T*. *trichiura* eggs between 30 and 60 minutes after slide preparation [17]. Each stool sample was then divided into two plastic containers labeled with the same identification number. One container was stored in a refrigerator at around 5°C. The other container was stored at room temperature at around 25°C. The following day, roughly 24 hours later, one Kato-Katz thick smear was prepared from each of these stool samples and analyzed as described above. The same process was repeated at 48 hours. Fecal egg counts of all STHs at 0, 24, and 48 hours from samples stored at room temperature and in a refrigerator were recorded by the laboratory technicians analyzing the Kato-Katz slides (Fig 2).

#### Homogenizing experiment

**Fig 3:**
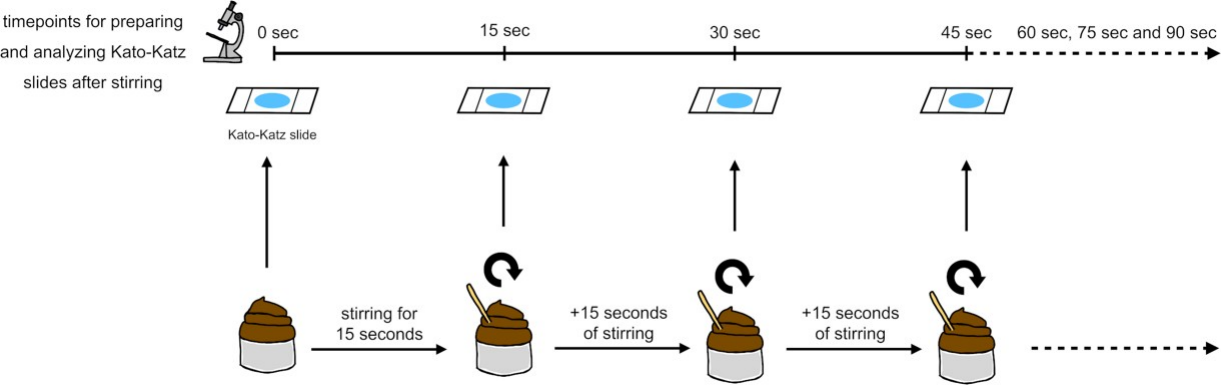
Procedure of the homogenizing experiment.

Stool samples selected for this experiment were not immediately homogenized. The pieces of stool used in this experiment were taken from unspecified regions of the stool samples, as usually done in routine analysis. First, a Kato-Katz thick smear was prepared from each sample and analyzed under a light microscope for hookworm, *A*. *lumbricoides* and *T*. *trichiura* eggs between 30 and 60 minutes after slide preparation [17]. Then each entire stool sample underwent 15 seconds of stirring with a wooden spatula. Again, a piece of stool was removed and used to prepare a Kato-Katz thick smear slide. This process of stirring and performing Kato-Katz thick smears took place six times for each whole stool sample. This resulted in seven Kato-Katz slides for each whole stool sample with FECs determined after 0, 15, 30, 45, 60, 75 and 90 seconds of homogenizing. A new wooden spatula was used at every time point. All Kato-Katz thick smears were microscopically analyzed for hookworm, *A*. *lumbricoides* and *T*. *trichiura* between 30 and 60 minutes after preparation (Fig 3).

### Statistical analysis

Stool samples with less than two eggs in the first Kato-Katz thick smear were not included in the study. Infections were classified as light, moderate or heavy according to WHO guidelines [33].

The Kato-Katz storing experiment was analyzed using mixed-effect models with Gaussian error terms and a random subject effect to account for repeated measurements. We modeled the logarithm of the FECs +1 as approximately normal, which resulted in a substantially better model fit compared to Poisson or negative binomial models. Time was included as a continuous variable and, therefore, we included only the equally spaced time intervals in the analysis for the Kato-Katz storing experiment, *i*.*e*. the observations up to 140 minutes, which were all 20 minutes apart. Besides storage type and time, the storage x time interaction was included for the Kato-Katz storing experiment. The whole sample storing experiment was analyzed by a paired t-test using the log transformed FECs as the outcome. Confidence intervals (CIs) for relative changes in medians and means were estimated using bootstrap resampling techniques with 5000 replications. For the homogenizing experiment, our primary interest was to assess if the variation of FECs decreases with increased stirring time. The change in variability was estimated using a two-step analysis. First, we calculated the relative difference of each observation from the overall mean FEC per stool sample. Because light infections are especially prone to relative changes, only samples with FECs of five or more at the first time point were included in the analysis. Thereafter we fitted a random effect linear regression model with stirring time as a predictor to estimate the residuals. Finally, we used the absolute values of the residuals as an outcome and fitted again a random effect regression model to quantify the change in variation. This approach has the advantage that it accounts for potential changes in mean FECs with increasing stirring time. All analyses were conducted using the statistical software environment R version 3.5.1.

## Results

### Sample size and infection intensities

A total of 92 samples were used in the Kato-Katz storing experiment, 340 in the whole sample storing experiment and 56 in the homogenizing experiment. The number of hookworm, *A*. *lumbricoides*, and *T*. *trichiura* infections and the respective infection intensities observed in each experiment are summarized in Table 1.

**Table 1:** Sample size, number of infections and infection intensities by experiment and parasite based on the first Kato-Katz thick smear.

			Kato-Katz storing experiment				Whole sample storing experiment		Homogenizing experiment	
			Room		Refrigerator					
			n	%	n	%	n	%	n	%
**Total samples**			92	100	92	100	340	100	56	100
**Total infections with**										
	Hookworm		67	72.8	62	67.4	259	76.2	28^a^	50.0
	*Ascaris lumbricoides*		38	41.3	38	41.3	163	47.9	19^a^	33.9
	*Trichuris trichiura*		85	92.4	85	92.4	313	92.1	52^a^	92.9
**Single infections**										
	Hookworm		6		3		18		0	
	*Ascaris lumbricoides*		0		0		7		1	
	*Trichuris trichiura*		15		17		31		10	
**Co-infections**										
	Hookworm- *Ascaris lumbricoides*		0		0		2		0	
	Hookworm- *Trichuris trichiura*		32		30		128		26	
	*Ascaris lumbricoides*—*Trichuris trichiura*		9		9		43		7	
	All 3 parasites		29		29		111		12	
**Infection intensities**										
	Hookworm									
		Light	65	97	59	95.2	246	95	23	81.9
		Moderate	1	1.5	2	3.2	11	4.2	3	10.7
		Heavy	1	1.5	1	2.6	2	0.8	2	7.4
	*Ascaris lumbricoides*									
		Light	14	36.8	15	39.5	62	38	3	15.8
		Moderate	24	63.2	23	60.5	81	49.7	14	73.7
		Heavy	0	0	0	0	20	12.3	2	10.5
	*Trichuris trichiura*									
		Light	50	58.8	55	64.7	200	63.9	23	44.1
		Moderate	35	41.2	29	34.1	111	35.5	28	53.9
		Heavy	0	0	1	1.2	2	0.6	1	2

a Only samples with fecal egg counts of at least five at the first measurement were included.

### Kato-Katz storing experiment

**Fig 4:**
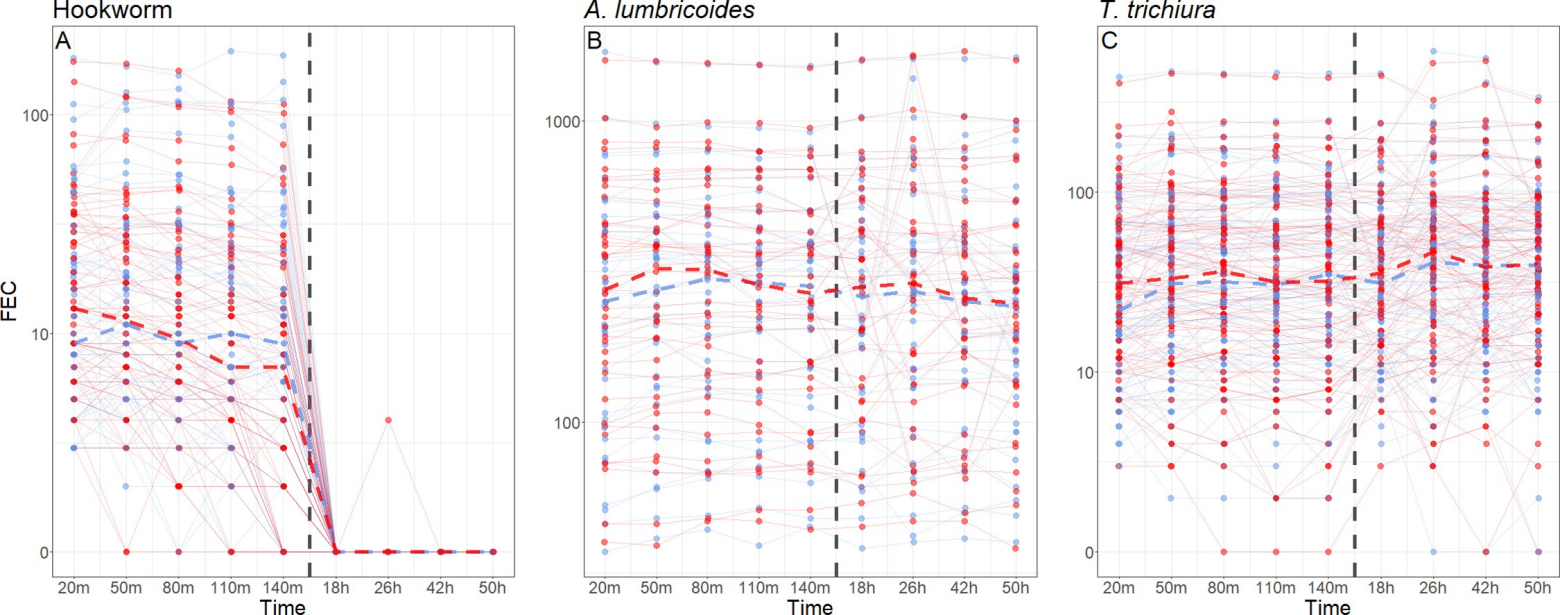
Kato-Katz storing experiment: Absolute median fecal egg counts (FEC) change over time for (A) hookworm (n = 67 for room temperature, n = 62 for refrigerator), (B) *A*. *lumbricoides* (n = 38) and (C) *T*. *trichiura* (n = 85). Blue = refrigerator temperature, red = room temperature, *A*. *lumbricoides* = *Ascaris lumbricoides*, *T*. *trichiura* = *Trichuris trichiura*.

Storing Kato-Katz slides at room temperature (n = 67), resulted in a steady decline in mean hookworm FECs from 21.7 to 13.1 between 20 and 140 minutes (Table 2). In addition, median FECs decreased from 12.0 to 10.5 after 50 minutes (relative change in medians -12.5%, 95% CI: -0.27 to 0.22), 8.5 after 80 minutes (-29.2%, 95% CI: -0.40 to 0.00), and 6 after 110 minutes (-50%, 95% CI: -0.60 to -0.12). The classification of samples as positive or negative, light or moderate/heavy was also influenced by the time between the slide’s preparation and reading. After 50 minutes, 4% of the samples kept at room temperature went from being hookworm-positive to -negative. In contrast, when storing slides in the refrigerator (n = 62), no noteworthy reduction in mean hookworm FECs was observed over the same time period (19.4 vs 19.7). A test for storage×time interaction revealed that the reduction in FECs over time was significantly higher in samples stored at room temperature compared to samples stored in the refrigerator (coefficient_log egg counts_: 0.26, 95% CI: 0.17 to 0.35, p < 0.0001; S1 Table). After 18 hours, regardless of the storing condition, FECs dropped to virtually zero (Table 2 and Fig 4A).

**Table 2:** Kato-Katz storing experiment: Results for hookworm, *Ascaris lumbricoides* and *Trichuris trichiura*. FECs = fecal egg counts, *A*. *lumbricoides* = *Ascaris lumbricoides*, *T*. *trichiura* = *Trichuris trichiura*, min = minutes, h = hours.

		Time of storing								
	Storing condition	20 min	50 min	80 min	110 min	140 min	18 h	26 h	42 h	50 h
**Hookworm**										
Mean FECs	Room (n = 67)	21.7	21.4	18.7	15.8	13.1	0.0	0.0	0.0	0.0
	Refrigerator (n = 62)	19.4	22.2	21.8	21.1	19.7	0.0	0.0	0.0	0.0
Median FECs	Room	12.0	10.5	8.5	6.0	6.0	0.0	0.0	0.0	0.0
	Refrigerator	8.0	10.0	8.0	9.0	8.0	0.0	0.0	0.0	0.0
***A*. *lumbricoides***										
Mean FECs	Room (n = 38)	374.7	382.4	383.9	371.78	362.8	363.0	401.5	351.7	339.7
	Refrigerator (n = 38)	350.5	366.7	363.8	358.1	356.5	352.6	386.2	347.4	336.7
Median FECs	Room	287.0	331.0	328.0	290.0	282.0	288.0	289.0	261.0	259.0
	Refrigerator	259.5	280.0	300.0	293.0	305.0	269.0	274.0	260.0	245.0
***T*. *trichiura***										
Mean FECs	Room (n = 85)	50.0	57.2	53.8	54.0	53.2	53.6	62.8	62.6	55.3
	Refrigerator (n = 85)	42.1	49.3	53.9	53.4	53.2	51.6	62.0	62.4	55.8
Median FECs	Room	30.0	32.0	35.0	30.5	31.0	34.5	45.5	37.0	39.0
	Refrigerator	21.0	30.0	30.5	29.5	34.0	30.0	39.5	38.0	38.0

For *A*. *lumbricoides* no consistent trend over time was observed in the mean FECs of slides stored at room temperature (n = 38) or in the refrigerator (n = 38) (Table 2 and Fig 4B). The storage×time interaction was statistically significant, but the effect size was quite small (coefficient_log egg counts_: 0.03, 95% CI: 0.00 to 0.06, p = 0.04; S1 Table).

In the case of *T*. *trichiura*, mean FECs of both storage types (n = 85) remained mostly stable but increased at the 26 hours timepoint (Table 2 and Fig 4C). The storage×time interaction indicated that slides stored in the refrigerator had significantly smaller reduction in FECs over time than those at room temperature (coefficient_log egg counts_ = 0.17, 95% CI: 0.12 to 0.23, p < 0.0001; S1 Table). Of note, although the coefficient for *T*. *trichiura* (0.17) is lower than that of hookworm (0.26), the relative effect is less pronounced for *T*. *trichiura* because the mean FECs are two times higher (Table 2). For all three parasites, if slides were kept in the refrigerator, FECs increased from the 20 to the 50 minutes time point (Table 2 and Fig 4).

### Whole sample storing experiment

**Fig 5:**
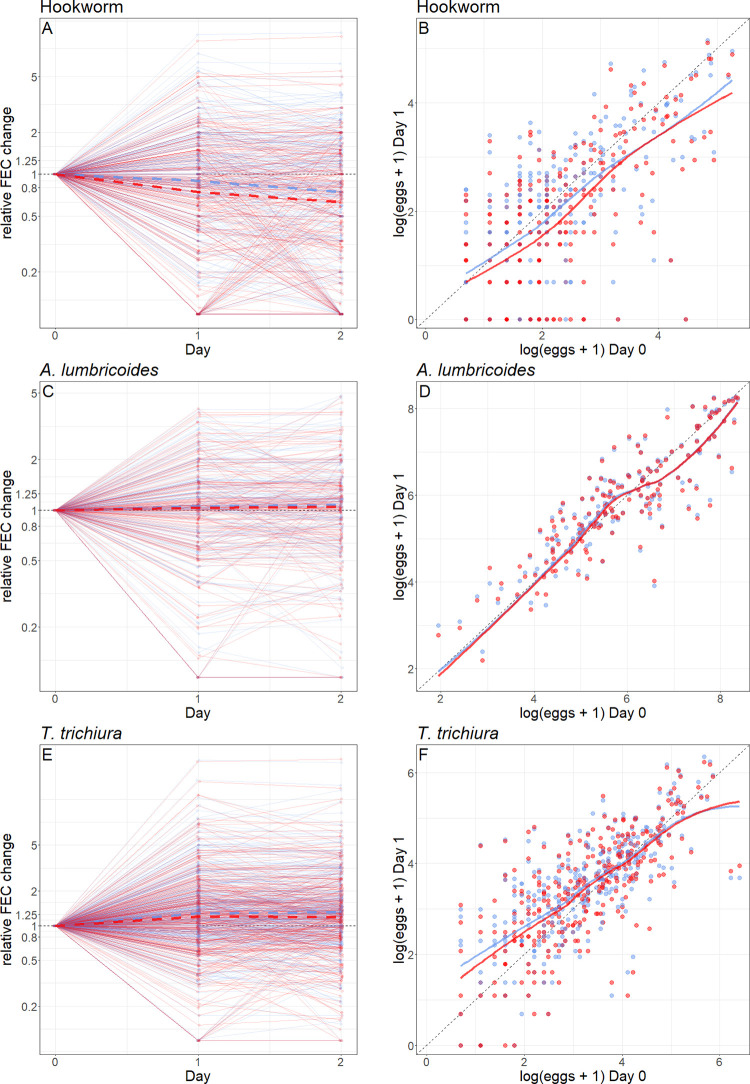
**Whole sample storing experiment: Relative fecal egg counts (FEC) change over time for (A) hookworm (n = 259), (C) *Ascaris lumbricoides* (n = 163), (E) *Trichuris trichiura* (n = 313) and absolute FEC numbers and relative FEC change between day 0 and day 1 for hookworm (B), *Ascaris lumbricoides* (D) and *Trichuris trichiura* (F).** For A, C and E: Dashed line = median. For B, D and F: Line = mean. Blue = refrigerator temperature, red = room temperature, *A*. *lumbricoides* = *Ascaris lumbricoides*, *T*. *trichiura* = *Trichuris trichiura*.

Storing hookworm stool samples in the refrigerator resulted in a lower FEC change compared to storing them at room temperature. For hookworm samples (n = 259) stored at room temperature, mean FECs decreased by 23% from 19.2 at baseline to 14.8 after 24 hours (95% CI: 0.12 to 0.33, p < 0.0001), compared to a reduction of 13% (mean FEC after 24 hours: 16.7; 95% CI: -0.01 to 0.24, p < 0.0001) for samples stored in the refrigerator (Table 3 and Fig 5A). The mean FEC change among storage conditions was statistically significant (mean difference after 24 hours: 1.9 FECs, p < 0.0001). A similar picture was observed after 48 hours (difference: 2.1 FECs, p < 0.0001; S2 Table). The classification of hookworm samples as positive or negative, light or moderate/heavy was influenced by storing samples overnight. If stored at room temperature, 17% of hookworm samples changed from positive to negative from day 0 to day 1, whereas only 13% of those stored in the refrigerator underwent this change. However, hookworm-positive samples stored in the refrigerator were not any less likely to change from being classified as moderate/heavy to light from day 0 to day 1 or from day 0 to day 2, compared to those stored at room temperature.

**Table 3:** Whole sample storing experiment: Mean FECs of day 0, day 1 and day 2 for hookworm, *A*. *lumbricoides* and *T*. *trichiura*. Rel change = relative change, FECs = fecal egg counts, *A*. *lumbricoides* = *Ascaris lumbricoides*, *T*. *trichiura* = *Trichuris trichiura*.

	Storing condition	Day 0	Day 1	Day 2	Rel change 0 vs 1 (%)	p-value	Rel change 0 vs 2 (%)	p-value
**Hookworm**								
**Mean FECs**	Room (n = 259)	19.2	14.8	14.8	-22.7	< 0.0001	-22.8	< 0.0001
	Refrigerator (n = 259)	19.2	16.7	16.8	-12.9	< 0.0001	-12.1	< 0.0001
***A*. *lumbricoides***								
**Mean FECs**	Room (n = 163)	754.0	687.5	704.1	-8.8	0.11	-6.6	0.43
	Refrigerator (n = 163)	754.0	698.4	711.0	-7.4	0.21	-5.7	0.65
***T*. *trichiura***								
**Mean FECs**	Room (n = 313)	49.6	58.5	59.7	17.9	< 0.0001	20.4	< 0.0001
	Refrigerator (n = 313)	49.6	61.1	62.0	23.2	< 0.0001	24.9	< 0.0001

The *A*. *lumbricoides* samples (n = 163) stored at room temperature showed a non-significant reduction in FECs of 9% from 754 at baseline to 688 after 24 hours, compared to a non-significant reduction of 7% (mean FEC after 24 hours: 698) in samples stored in the refrigerator (Table 3). Although the difference among the two storing conditions was statistically significant, it was negligible (mean difference after 24 hours: 10.9 FECs, p = 0.01; mean difference after 48 hours: 6.9 FECs, p = 0.05; S2 Table).

In the case of *T*. *trichiura* mean FECs in samples (n = 313) stored at room temperature increased significantly from day 0 to day 1 (by 18% from 49.6 at baseline to 58.5 after 24 hours, p < 0.0001) and from day 0 to day 2 (by 20%, mean FECs after 48 hours: 59.7, p < 0.0001). Similar results were observed for samples stored in the refrigerator (Table 3 and Fig 5E). The mean FEC change between samples stored at room and refrigerator temperature was statistically significant, but it was very slight with about 4% (mean difference after 24 hours: 2.59 FECs, p < 0.0001; mean difference after 48 hours: 2.23 FECs, p < 0.0001; S2 Table).

The relative change of FECs from one day to the next seems to be influenced by infection intensities. In the case of hookworm and *A*. *lumbricoides*, higher infection intensities lead to a higher mean relative FEC change irrespective of the storing condition (Fig 5B and 5D). The same was found for *T*. *trichiura* but, in addition to that, light *T*. *trichiura* infections resulted in higher FECs the next day (Fig 5F).

### Homogenizing experiment

**Fig 6:**
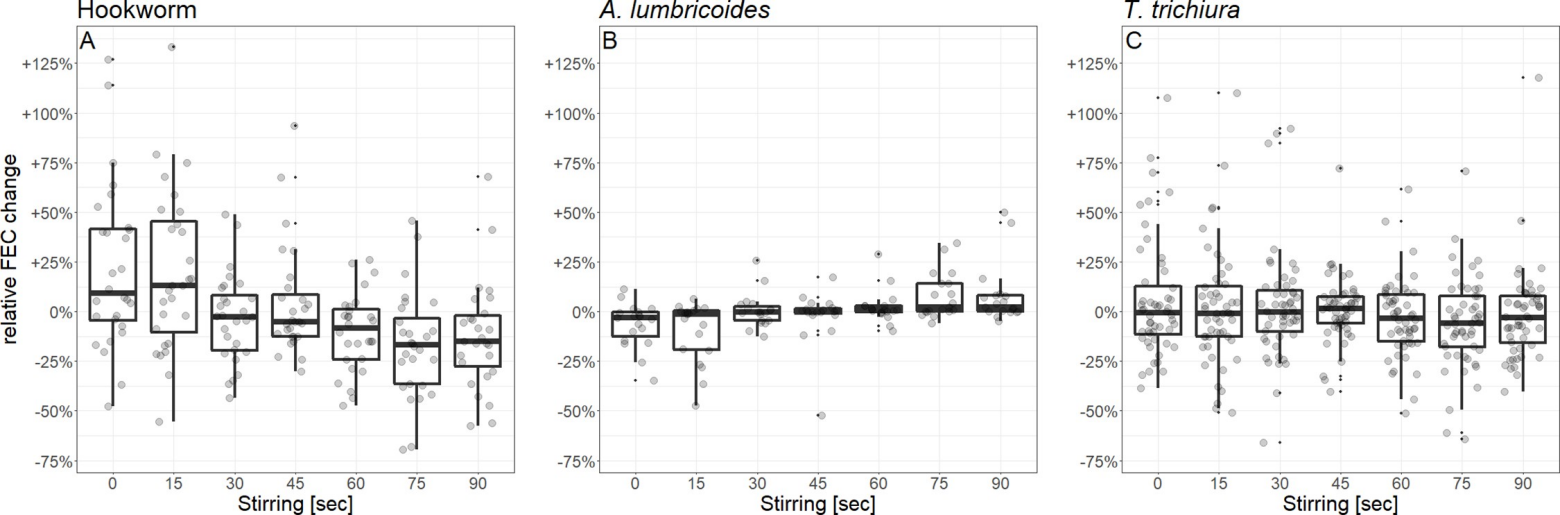
**Homogenizing experiment: Relative fecal egg counts (FEC) differences from the overall mean per sample (over 7 observations) after different lengths of homogenization for (A) hookworm (n = 28), (B) *A*. *lumbricoides* (n = 19) and (C) *T*. *trichiura* (n = 52).** Figures show boxplots and observed values (jittered). *A*. *lumbricoides* = *Ascaris lumbricoides*, *T*. *trichiura* = *Trichuris trichiura*.

With increasing rounds of stirring, mean FECs decreased in samples with hookworm eggs (n = 28; Fig 6A and Table 4). For *A*. *lumbricoides* (n = 19) we observed a slight increase in mean FECs with every 15 seconds of extra stirring, whereas FECs in *T*. *trichiura* samples (n = 52) remained constant (Fig 6B and 6C and Table 4). The statistical analysis of the residuals showed a statistically significant reduction of variation of FECs for hookworm samples (change in residuals per 15 seconds: -0.02, 95% CI: -0.03 to -0.007, p = 0.004) and *T*. *trichiura* samples (change per 15sec: -0.01, 95% CI: -0.02 to -0.002, p = 0.01), but not for *A*. *lumbricoides* samples (change per 15sec: -0.001, 95%CI: -0.007 to 0.005, p = 0.66). The graphical inspection of the boxplots revealed that for *T*. *trichiura* samples stirring had, in the first case, an impact on the number of extreme values but the width of the interquartile range was less affected (Fig 6C).

**Table 4:** Homogenizing experiment: Mean fecal egg counts (FECs), mean relative differences from the grand mean FECs per sample (over 7 observations), standard deviation of the relative differences and mean values of the residuals estimated from the random effect regression model. *A*. *lumbricoides* = *Ascaris lumbricoides*, *T*. *trichiura* = *Trichuris trichiura*.

	Stirring time (seconds)						
	0	15	30	45	60	75	90
**Hookworm (n = 28)**							
Mean FECs	51	52	47	48	46	46	46
Relative difference from grand mean	1.22	1.20	0.97	1.04	0.90	0.83	0.85
Standard deviation of relative difference	0.34	0.35	0.23	0.23	0.23	0.36	0.42
Mean absolute residual	0.32	0.31	0.19	0.18	0.16	0.20	0.22
***A*. *lumbricoides* (n = 19)**							
Mean FECs	695	707	732	722	734	747	749
Relative difference from grand mean	0.93	0.90	1.01	0.98	1.02	1.08	1.08
Standard deviation of relative difference	0.13	0.19	0.08	0.18	0.08	0.10	0.12
Mean absolute residual	0.09	0.12	0.06	0.06	0.05	0.09	0.10
***T*. *trichiura* (n = 52)**							
Mean FECs	80	81	81	79	85	76	76
Relative difference from grand mean	1.06	1.02	1.03	1.01	0.97	0.94	0.98
Standard deviation of relative difference	0.26	0.28	0.27	0.17	0.21	0.28	0.21
Mean absolute residual	0.21	0.21	0.18	0.11	0.14	0.17	0.15

## Discussion

The Kato-Katz technique is the current mainstay for the diagnosis of STH infections and it is recommended by the WHO for its simplicity and cost-effectiveness [2,9–11,17]. However, although it is highly sensitive in detecting moderate and heavy infection intensities, its sensitivity to light infections is quite low, especially for hookworm, since hookworm eggs are fairly unstable [2,22]. Although the Kato-Katz method has been used for several decades, the influence of factors like storage time, storage temperature, and stirring of stool samples, has not been explored in depth.

### How long can Kato-Katz slides be stored at different temperatures before analysis?

To avoid the clearing of hookworm eggs, the WHO recommends reading a Kato-Katz thick smear 30 to 60 minutes after the slide is prepared [17]. Although the effect of time between the preparation of slides and reading them on hookworm FECs has been investigated [28] or debated [2,9,26] in several studies, the time period during which hookworm FECs remain visible on Kato-Katz slides and whether storing them in a refrigerator would preserve them for longer had never been studied. We found that for hookworm, FECs started decreasing from 20 minutes after slide preparation. In our study, if kept at room temperature, the WHO’s recommendation of reading slides up to 60 minutes led to the clearing of around 10% of hookworm eggs before reading. The experiment also revealed that storing Kato-Katz slides in the refrigerator had a positive influence on preserving hookworm eggs up to 110 minutes, hence storing temperature can play an important role. Our results are in line with a study from the 1960s; the authors exposed slides to 24, 30 and 36°C and found that hookworm eggs cleared faster with increasing temperatures [28].

In contrast to hookworm, *T*. *trichiura* FECs increased after 26 hours. This unexpected result could have different explanations. A first one might be related to the staining of eggs; perhaps the longer one waits, the more visible *T*. *trichiura* eggs become. A second explanation is that the natural variation in egg counting or the reader variability might have influenced the results, since the slides were not assigned randomly to laboratory technicians and they were not blinded. Further studies should explore these two issues in more detail. For *A*. *lumbricoides*, storage of Kato-Katz thick smears for up to 50 hours did not influence FECs of Kato-Katz slides. Furthermore, for *A*. *lumbricoides* and *T*. *trichiura*, there was only a slight difference between storing conditions. Thus, in settings with electricity and available refrigerators, we recommend to store Kato-Katz slides in a refrigerator to avoid an underestimation of FECs.

Interestingly, slides stored in the refrigerator had a slight FEC increase after 50 minutes, for all three parasites. We presume that storing the Kato-Katz slides in the refrigerator immediately after preparation slowed down the STH egg staining process. Hence, after 20 minutes of being stored in the refrigerator, some eggs were still invisible to laboratory technicians and, therefore, not recorded. It is, therefore, important to avoid placing slides in the refrigerator immediately after slide preparation. Instead this should only be done 20 minutes later.

Our findings revealed that when slides are stored at room temperature (around 25°C), hookworm FECs decay even faster with a 30% decrease of median FECs after 80 minutes since slide preparation. Our findings might contribute to an adapted Kato-Katz technique to preserve hookworm eggs for longer.

### What is the effect of storage time and storage temperature on whole stool sample’s FECs?

Hookworm eggs have been described to quickly disappear in stool samples [18,22,23]. We thoroughly investigated the effect of storage time and storing temperature on FECs in whole stool samples. Our findings show that, at room temperature, there is a significant decrease of hookworm FECs from collection day to the next day, as well as from collection day to the second day. Although the decrease of FECs was also observed when samples were stored in a refrigerator, the decrease was only half the size. No noteworthy differences were observed between one day or two days of sample storing. Krauth *et al*. found a significant FEC reduction in samples when they were kept for 8 hours at room temperature, but this was not the case when stored on ice or covered with a moist tissue [23]. This contradictory finding might be due to the different examination time points, as our first assessment was done later, 24 hours after sample collection. It is important to note that, in the case of hookworm, if stool samples are not analyzed on the same day, storing samples from one day to the next, regardless of the storing condition, can have an impact on the classification of samples as light or moderate/heavy intensities, and even as positive or negative. With increasing storing time, we observed a slight decrease in FECs for *A*. *lumbricoides* samples whereas, surprisingly, the median FECs of *T*. *trichiura* even increased from day 0 to day 1, and from day 0 to day 2 for both storing conditions. This might be related to natural variation in egg counting, to reader variability or to the fact that readers did not count the eggs as carefully on the two follow-up days, particularly when infection intensities were high, since counting all the eggs is laborious and time consuming.

Krauth and colleagues found no effect of time or temperature on *T*. *trichiura* FECs during the first 8 hours after sample collection. They do not present results for *A*. *lumbricoides* due to the small sample size. Interestingly, we observed that, for both parasites, FECs of samples stored in the refrigerator were significantly higher than those stored at room temperature. This could either be by chance or perhaps *A*. *lumbricoides* and *T*. *trichiura* eggs are more stable or visible when stored in a refrigerator.

### Does homogenizing stool samples change the variation of FECs?

Homogenizing stool samples by stirring them prior to the preparation of Kato-Katz slides has been recommended by some authors [18,22,31]. Although it is not specifically mentioned in the WHO bench aids for the diagnosis of intestinal parasites, homogenization of stool samples is often performed in studies working with STH [34]. In our current study, we aimed at determining whether this step influences the variation of FECs, and for how long one should stir the sample. We found different results for different species. Homogenizing samples before slide preparation significantly reduced the variation of FECs for hookworm and *T*. *trichiura* samples. For *T*. *trichiura* samples, stirring had an effect, especially on the frequency of extreme events, *i*.*e*. FECs with large deviations from the mean FEC. These findings are in line with those described by Coffeng *et al*., who state that stool homogenization reduced variation between different slides of the same stool sample [31]. In our study, no noteworthy effect was observed for samples of *A*. *lumbricoides*, but the sample size was relatively small.

However, for hookworm samples, we observed a reduction of FECs with increasing rounds of stirring. This was not the case for *A*. *lumbricoides* and *T*. *trichiura* FECs. It might be that, because hookworm eggs are particularly fragile, with increasing rounds of stirring, the thin-shelled ova of hookworm were destroyed and became invisible to laboratory technicians. This finding needs to be further investigated because, if confirmed, homogenizing samples with hookworm eggs could have important consequences, such as an underestimation of this parasite’s prevalence.

Previous studies reported that hookworm, *A*. *lumbricoides* and *T*. *trichiura* eggs are patchily distributed in stool samples [20,23,24,26,27,29]. Krauth *et al*. and Ye *et al*. found no significant difference in FECs or in the distribution of FECs (in the case of Ye *et al*.) analyzed in hookworm and *T*. *trichiura* samples taken from the surface and the center of the stool. Interestingly, Krauth *et al*. detected that hookworm FECs were significantly higher in the front piece of the stool sample than those in the back piece; the exact opposite was found for *T*. *trichiura* where FECs were significantly higher in the back piece, compared to the front piece of stool. These findings suggest an aggregation of eggs distributed along the length axis of a sample. However, these two studies did not find a reduction in variation of FECs in samples after homogenization (stirring for 60 seconds and as long as 15 to 20 minutes, in the case of Krauth *et al* and Ye *et al*., respectively) for *A*. *lumbricoides* and *T*. *trichiura* [24], or any of the three parasites [23]. This might be explained by the small sample size of these studies (ranging from two to nine). Also, Krauth *et al*. did not detect any change in FECs after homogenization for any of the three parasites [23].

Our results showed that, although homogenizing samples seems to decrease the variation of FECs, it might also lead to a reduction of hookworm FECs. Hence, it is unclear if homogenization actually increases the sensitivity of the Kato-Katz technique.

**Fig 7:**
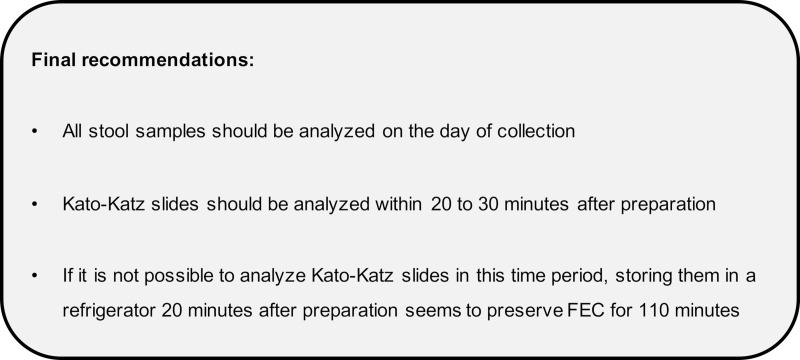
Final recommendations on the procedure of the Kato-Katz technique.

Our final recommendations concerning the Kato-Katz technique are presented in Fig 7.

### Strengths and limitations

Other studies in this field had very small sample sizes ranging from two to 14 whole stool samples [23,24,28]. Thus, a major strength of our study is the large sample size we had for all three experiments. Another strength of our study is that, because it took place in a highly endemic region for STH, many of our samples had moderate or heavy STH infection intensities, which was not the case for the other studies.

Our study had a few limitations. The first was that we did not record the exact time of defecation, which keeps us from knowing the exact period between sample production and sample analysis. Also, we did not measure the exact time from sample collection to laboratory processing, although they took place during the same morning. Future studies should record these time points, so they can be taken into account. Finally, we did not measure the time span needed to analyze each individual Kato-Katz slide, which would have been useful information to help interpret the Kato-Katz storing experiment. With that information we would have been able to know exactly for how long each slide was stored in the refrigerator between reading time-points. From our observations, we estimated that it took our laboratory technicians about eight minutes to read a Kato-Katz slide for all three parasites. This time span is in line with a publication from Speich *et al*., who estimated 9.5 minutes in average for reading a Kato-Katz slide for all three STH [12].

Another limitation is that the Kato-Katz thick smears were not randomly assigned to laboratory technicians. Also, because all three experiments required strict punctuality, Kato-Katz slides were not re-labelled with new identification numbers at each time point, meaning laboratory technicians were not blinded and we did not perform quality control.

A further limitation concerns the time points we chose for the whole sample storing experiment. It would have been interesting to investigate another time point later on day 0 (*e*.*g*. 8 hours) to investigate the effect of the time span between sample collection and analysis within the collection day on hookworm FECs. In the case of the Kato-Katz slide experiment, a 30-minute time point should have been considered, to match WHO recommendations.

A final limitation was that we did not take into account stool consistency and this could have some influence on results.

## Conclusions

Based on our results, we suggest analyzing Kato-Katz slides between 20 and 30 minutes after slide preparation and a revision of the WHO recommendations accordingly. If it is not possible to read Kato-Katz slides within this time period, we recommend that, 20 minutes after preparation, they are stored in a refrigerator since we found that this could delay the clearing of hookworm FECs up to 110 minutes. Also, our results showed that storing whole stool samples overnight leads to a significant reduction of hookworm FECs, regardless of the temperature at which they are stored. Therefore, we recommend analyzing all stool samples the day they are collected (Fig 7). Finally, we found that there is a significant reduction of variation in hookworm and *T*. *trichiura* FECs when whole stool samples are homogenized before Kato-Katz slide preparation. Since our results also showed that hookworm FECs decreased with increasing rounds of stirring, it is difficult to draw recommendations concerning sample stirring time. Future studies should further investigate the reason behind the reduction of FECs with homogenizing.

## Supporting information

S1 Table Kato-Katz storing experiment: Interaction model between time and storing condition. CI = confidence interval, FECs = fecal eggs counts, *A*. *lumbricoides* = *Ascaris lumbricoides*, *T*. *trichiura* = *Trichuris trichiura*.
(DOCX)

S2 Table Whole sample storing experiment: Difference of mean FECs between storing conditions for hookworm, *A*. *lumbricoides* and *T*. *trichiura*. FECs = fecal egg counts, *A*. *lumbricoides* = *Ascaris lumbricoides*, *T*. *trichiura* = *Trichuris trichiura*.
(DOCX)
