# Supplementary material for: Diagnosis of soil-transmitted helminths using the Kato-Katz technique: What is the influence of stirring, storage time and storage temperature on stool sample egg counts?
Source: PLoS Negl Trop Dis. 2021 Jan 22;15(1):e0009032. doi: 10.1371/journal.pntd.0009032 (PMC7857572; doi:10.1371/journal.pntd.0009032)
Supplement: S1 Table — CI = confidence interval, FECs = fecal eggs counts, A. lumbricoides = Ascaris lumbricoides, T. trichiura = Trichuris trichiura. (DOCX) [file pntd.0009032.s001.docx]

|  | **Hookworm (n = 62)** | | | | ***A. lumbricoides* (n = 38)** | | | | ***T. trichiura* (n = 85)** | | | |
| --- | --- | --- | --- | --- | --- | --- | --- | --- | --- | --- | --- | --- |
|  | **log egg count** | **95% CI** | | **p-value** | **log egg count** | **95% CI** | | **p-value** | **log egg count** | **95% CI** | | **p-value** |
| Intercept | 2.77 | 2.51 | 3.03 | < 0.0001 | 5.63 | 5.35 | 5.90 | < 0.0001 | 3.52 | 3.28 | 3.75 | < 0.0001 |
| Change in FECs per hour: both storing conditions | -0.42 | -0.48 | -0.36 | < 0.0001 | -0.02 | -0.04 | 0.01 | 0.18 | -0.04 | -0.08 | -0.01 | 0.03 |
| Change in FECs per hour: refrigerator | -0.31 | -0.45 | -0.17 | < 0.0001 | -0.09 | -0.14 | -0.04 | 0.0002 | -0.31 | -0.39 | -0.23 | < 0.0001 |
| Interaction of storage type (room temperature) and time (1 hour) | 0.26 | 0.17 | 0.35 | < 0.0001 | 0.03 | 0.00 | 0.06 | 0.04 | 0.17 | 0.12 | 0.23 | < 0.0001 |

**S1 Table**
