# Supplementary material for: Diagnosis of soil-transmitted helminths using the Kato-Katz technique: What is the influence of stirring, storage time and storage temperature on stool sample egg counts?
Source: PLoS Negl Trop Dis. 2021 Jan 22;15(1):e0009032. doi: 10.1371/journal.pntd.0009032 (PMC7857572; doi:10.1371/journal.pntd.0009032)
Supplement: S2 Table — FECs = fecal egg counts, A. lumbricoides = Ascaris lumbricoides, T. trichiura = Trichuris trichiura. (DOCX) [file pntd.0009032.s002.docx]

**S2 Table.**

|  | **Hookworm (n = 259)** | | ***A. lumbricoides* (n = 163)** | | ***T. trichiura* (n = 313)** | |
| --- | --- | --- | --- | --- | --- | --- |
|  | **FECs** | **p-value** | **FECs** | **p-value** | **FECs** | **p-value** |
| Difference of mean FECs between storing conditions from day 0 to day 1 | 1.88 | < 0.0001 | 10.88 | 0.01 | 2.59 | < 0.0001 |
| Difference of mean FECs between storing conditions from day 0 to day 2 | 2.06 | < 0.0001 | 6.94 | 0.05 | 2.23 | < 0.0001 |
